# A Child With Hereditary Spherocytosis Associated With Von Willebrand’s Disease: A Case Report From Saudi Arabia

**DOI:** 10.7759/cureus.29733

**Published:** 2022-09-29

**Authors:** Mohammed A Zolaly, Mariam M Alemam, Omar M Kheder, Abdullah K Aljohani

**Affiliations:** 1 Hematology and Oncology, Taibah University, Medina, SAU; 2 Pediatrics, Taibah University, Medina, SAU; 3 Hematology, Prince Mohammed Bin Abdulaziz Hospital, Medina, SAU; 4 Medical Student, Taibah University, Medina, SAU

**Keywords:** child, saudi arabia, hemolytic anemia, consanguinity, hereditary spherocytosis, von willebrand’s disease

## Abstract

Von Willebrand disease (VWD) is an autosomal inherited hemostasis disorder caused by a deficiency or defect in the blood protein known as von Willebrand factor, which is necessary for platelets to adhere to damaged vessel walls. The main symptoms of the condition include spontaneous bleeding from mucosal membranes, excessive wound bleeding, and menorrhagia in girls. On the other hand, hereditary spherocytosis (HS) is a heterogeneous group of diseases that damage red blood cells, with clinical manifestations depending on the different membrane protein-encoding gene mutations, their different functional consequences, and the mechanism of inheritance. It is typically characterized by the presence of jaundice, anemia, and splenomegaly. Here, we report a novel pathogenic mutation in a child with HS that led to hemolytic anemia since the age of two years associated with recently discovered type 1 VWD, as we were unable to find any cases that have been previously reported to have HS associated with VWD. According to our analysis of the literature, there is no definitive link between the two hematological disorders.

## Introduction

The most common type of congenital hemolytic anemia caused by a defective red cell membrane is hereditary spherocytosis (HS). The genetic mutation in erythrocyte membrane proteins results in losing their typical biconcave shape. In northern Europe and northern America, it is widespread and affects one in every 2,000 individuals. Patients with HS usually present with a triad of anemia, splenomegaly, and jaundice which is common in older children and adults but is rare in neonates. The diagnosis is based on history, clinical presentation, and supporting laboratory findings, which include complete blood count (CBC), blood smear, reticulocyte count, bilirubin levels, and red cell osmotic fragility test along with confirmatory genetic testing.

The main goals of the treatment plan are to enhance the quality of life of the patient, prevent expected complications of HS, and adequately treat them when present [1,2].

It is well-known that the most common autosomal inherited bleeding disease is von Willebrand disease (VWD) with an incidence of 1 in 1,000 people worldwide. The disease is caused by a defect in von Willebrand factor (VWF) which is a crucial glycoprotein in normal hemostasis. The disease is classified into three types according to its severity and primary cause. In type 1, the deficiency is a partial quantitative defect, type 2 is a qualitative defect with different subtypes, and type 3 is a complete deficiency of the factor [3].

For clinical diagnosis, the clinical history is crucial, supported by laboratory tests, which include screening coagulation assays, followed by VWF testing to confirm the deficiency, as well as structural and functional assays to characterize the subtype of the factor in case of functional abnormalities [4].

Tranexamic acid, 1-deamino-8-d-arginine vasopressin (DDAVP), plasma-derived VWF concentrates, and recombinant VWF are available as therapy options when needed [5].

## Case presentation

A 13-year-old Saudi boy presented to the Department of Pediatrics Hematology in November 2020 with a history of anemia and complaints of shortness of breath after moderate activities. He was treated occasionally in the past with salbutamol for a wheezy chest which improved over time. At two years of age, he was diagnosed with anemia of unknown etiology as he presented with low hemoglobin and jaundice at a different medical institute.

On genetic testing conducted two years ago in Becenti Laboratories in Germany using next-generation sequencing (NGS), a heterozygous variant was identified in the c.281G>A p. (Trp94*) in the *SLC4A1* gene, which led to a pre-mature stop code and subsequent mRNA degradation (nonsense-mediated decay) or truncation of the protein. The *SLC4A1 *gene in our patient showed a novel mutation, and his family was referred to genetic counseling; however, we were unable to test the father as he lived in another city. The screening for both the mother and his brother was normal.

In the past, he had experienced episodes of recurrent easy fatigability, pallor, and jaundice, in addition to dark urine stained by urobilinogen. He was previously labeled as a case of iron deficiency anemia and received oral iron treatment on several occasions; however, his hemoglobin did not significantly improve.

He received blood transfusions twice for his anemic symptoms and low hemoglobin levels of less than 7 g/dL, but neither had any effect on the outcome nor provided a conclusive treatment. Two years ago, he began taking folic acid supplements, which caused him to feel better after his diagnosis of HS was confirmed.

His mother disclosed a history of iron-deficient anemia, but she never underwent blood replacement therapy. Moreover, she denied any family history of blood problems or splenectomy, and the patient’s brother was normal. There was no evidence of any other hematological disorders in the extended family members apart from a family history of VWD which was discovered during follow-up, and for which his history was revised again and revealed occasional attacks of mild epistaxis from both nostrils but never requiring emergency room admission. The patient underwent further investigations, including a complete blood count (CBC) (Table 1) and blood coagulation profile (Table 2), which showed a low VWF antigen level. Hence, he was diagnosed with type 1 VWD in addition to his primary disease of HS.

**Table 1:** Complete blood count and differential. WBC: white blood cell; Hgb: hemoglobin; RBC: red blood cell; MCV: mean corpuscular volume; MCH: mean corpuscular hemoglobin; MCHC: mean corpuscular hemoglobin concentration; RDW: red cell distribution width

Test	Results	Normal range
WBC	4.9	4–15.5 × 10³/µL
Hgb	13.6	12–16 g/L
RBC	4.36	4.3–5.7 million/µL
Hct	37	38–80%
Platelets	202	150–400 × 10³/µL
MCV	85.2	68–79 fL
MCH	31.2	19–28 pg
MCHC	36.6	30–38 g/dL
RDW	15	11–15%
Reticulocytes	6.58	0.5–1.5%
Absolute reticulocyte count	261	10–110 × 10⁹/L
Neutrophils	47.6	40–74%
Lymphocytes	34.4	14–46%

**Table 2:** Blood coagulation findings. VWF: von Willebrand factor; vWF:RCo: von Willebrand ristocetin cofactor; INR: international normalized ratio; PT: prothrombin time; PTT: partial thromboplastin time

Test	Results	Normal range
Factor Xlll	74	80–150%
VWF antigen	20	50–150%
VWF: RCo	28	50–150%
Factor Vlll	63	55–145%
INR	1.22	0.8–1.1
PT	14.40	11.0–12.5 seconds
PTT	27.30	60–70 seconds

Further investigations showed that his Coombs test was negative, and hemoglobin electrophoresis revealed hemoglobin A 97.8%, hemoglobin A2 2.2% (normal up to 2.5%). His peripheral blood smear showed many spherocytes with no abnormalities in other cell lines. His repeated laboratory investigation included liver function tests (Table 3) which revealed a picture of indirect hyperbilirubinemia, while his urea, creatinine, electrolytes, and blood gases were within normal limits.

**Table 3:** Liver function test findings.

Test	Results	Normal range
Aspartate aminotransferase	26	10–43 U/L
Aminotransferase	12	10–100 U/L
Alkaline phosphatase	279	24–147 U/L
Total bilirubin	39.8	1.71–20.5 μmol/L
Direct bilirubin	13.2	0–5.1 μmol/L

In December 2020 he was admitted to the hospital with hematuria and mild abdominal pain. Subsequently, he presented to his regular follow-up visits with sudden-onset left lower quadrant pain, stabbing in character, not radiating, relieved by simple pain killers and no aggravating factors, mild in severity, and no other associated symptoms. His coagulation profile was slightly prolonged, while his urine analysis showed moderate urobilinogen.

Physical examination showed a well-appearing child with stable vital signs and no overtly dysmorphic traits. He was alert, pale, and affected by slight scleral jaundice. His spleen was palpable 3 cm below the costal margin, and he had no lymphadenopathy. The liver span was 10 cm with a border barely palpable below the right costal margin. A slight hemic murmur was audible above the aortic area, but his cardiac examination revealed normal heart sounds. He had a normal skin examination with no bruising or petechial rash. The central nervous system was grossly intact.

Ultrasound of the abdomen showed that the liver was mildly enlarged in size in correlation with age measuring 14.2 cm, with a homogenous parenchymal echo pattern without biliary dilatation, demonstrating a previously noted left hepatic lobe hypoechoic lesion measuring 1.16 × 1.01 cm (previously 0.91 × 0.84 cm) a year ago appearing more heterogeneous with some vascularity within it. The gallbladder was normal with no stones or mud seen within, the common bile duct was normal, and the spleen was mildly enlarged in size in correlation with age measuring 14.1 cm (previously 12.5 cm) showing homogenous texture with no focal lesions. Both kidneys were normal in site, size, and contour with normal renal parenchymal thickness and echogenicity. No stones or hydronephrosis were noted on either side. The pancreas and midline structures were partially obscured by gases. No free fluid was seen in the abdomen. Right inguinal lymph nodes were seen.

Our patient is currently being managed with oral folic acid 5 mg/day and tranexamic acid 500 mg/dose in case of bleeding.

## Discussion

Hereditary bleeding disorders (HBDs) are a group of diseases with variable frequency in different ethnic groups. In Saudi Arabia, a high rate of consanguinity is considered a significant independent risk factor for the high prevalence of different inherited disorders, including HBDs. Unfortunately, few studies have explored the prevalence of HBDs in Saudi Arabia [6]. Zolaly conducted a study among university students in Medina to assess bleeding tendency, which showed 52.6% had a history of bleeding tendency, with oral cavity bleeding (39.6%) and mucocutaneous bleeding (22.6%) being the most common symptoms reported. This indicated that the cause of these bleeding symptoms can be platelet disorders or VWD [7-9].

Our patient was initially diagnosed with anemia at two years of age when he experienced episodes of easy fatigability, pallor, splenomegaly, and jaundice, in addition to dark urine caused most likely by urobilinogen. The diagnosis of iron deficiency anemia is common, but it was not the case in our patient as he was treated with iron several times as a differential of his microcytic anemia. He was managed later with a folic acid supplement with regular follow-ups in the hematology clinic to monitor his hemoglobin levels along with liver function and ultrasound of the abdomen. After obtaining the history, he was found to have frequent attacks of epistaxis, for which he did not require any interventions. The patient underwent investigations that showed low von Willebrand assay.

VWD is an inherited autosomal dominant disorder in most patients and is considered the most common type of inherited bleeding disorder (IBD) worldwide affecting males and females equally. A population‐based study was done in Riyadh to determine the prevalence of VWD among adolescents, and it estimated the prevalence to be 1.5% [10].

Diagnosis of bleeding disorders based on the signs and symptoms is sometimes very challenging. Accuracy of the diagnosis is crucial for preventing long-term complications and for applying preventive measures and proper counseling to the family. Although there has been an evolution in genetic testing and counseling of families with HBDs, there is a need for more scientific advancements in clinical research regarding bleeding disorders.

Our case had a novel pathological mutation in the *SLC4A1 *gene of HS [11]. To our knowledge, it is the first time this new mutation is reported in the medical literature, with few cases reporting different novel mutations causing HS. Sánchez-López et al. reported another novel mutation in the *SLC4A1 *gene [12]. Zwieten et al. reported 15 different mutations in the *SLC4A1 *gene [13]. None of these mutations linked HS with other hematological diseases. Tang et al. and Raimondo et al. reported novel mutations in the *SLC4A1 *gene causing HS and distal renal tubular acidosis [14]. According to our literature review, we could not find any case with a specific genetic mutation reported which could link both HS and VWD.

To our knowledge, there is no clear association between the two hematological disorders and the novel mutation in our patient. In Saudi Arabia, it is not uncommon to have more than one hereditary disease due to the high level of consanguinity. We suggest that there could be a link between both diseases and hematologists should ask about bleeding history in patients with HS and perform a VWF assay if any suspicion is raised or in case of the presence of a family history of an inherited bleeding disorder, especially if the patient has the same reported mutation as in our case causing HS. Reporting these findings can help patients to be screened early to prevent possible complications.

## Conclusions

On a literature review, we could not find any case with genetic mutations reported before to be a cause of both HS and VWD. According to our literature review, there is no clear association between the two hematological disorders and the novel mutation seen in our patient. We suggest that patients with HS found to have the same genetic mutation as our patient be screened for VWD if they have bleeding symptoms or if there is a family history of hereditary bleeding tendency.
